# Rapid Immunization Scheme for Spouses of Individuals Estabilished as Hepatitis B Carriers during Premarital Tests

**DOI:** 10.1155/2012/843134

**Published:** 2012-12-13

**Authors:** Selma Tosun, Murat Yücetürk, Aydın Bilal Dönmez, Turan Gündüz

**Affiliations:** ^1^Department of Clinical Microbiology and Infectious Diseases, Manisa State Hospital, Manisa, Turkey; ^2^Primary Health Care Clinic No. 2, Turgutlu, Manisa, Turkey; ^3^Department of Microbiology, Vocational School of Health Services, Celal Bayar University, Manisa, Turkey

## Abstract

*Background*. The aim of this study was to monitor the cases identified as hepatitis B carriers during premarital tests, to vaccinate their prospective spouses with a rapid vaccination scheme, and to compare the anti-HBs responses with the traditional vaccination scheme. *Methods*. Blood samples of 1250 couple spouses were tested for HBsAg and anti-HBs. HBsAg positive cases' fiancées which were found HBV negative were administered a rapid three-dose vaccination scheme on days 0, 7, and 21. Forty controls with similar age and gender were also were administered three doses of the same vaccine. *Results*. Out of 1250 cases (625 couples), 46 (3.6%) were HBsAg positive, and 40 of them aged between 18 and 39 were admitted to the rapid vaccination program. *Conclusion*. Upon determination of HBsAg positivity in premarital tests, a rapid vaccination program provides early protection, but the 6th and 12th month vaccinations are also required. Anti-HBs response should be monitored.

## 1. Introduction

As sexual intercourse is an important route of transmission for HBV, in case of the determination of a carrier status during premarital testing, protection of the prospective spouse by early vaccination is imperative [1]. Since both vertical and horizontal transmission is possible in HBV and children of HBsAg-positive mothers predominantly infected during delivery, the testing of pregnant women during pregnancy or even prior to marriage for carrier status is critical [2, 3].

The civil law in effect as of 2002 suggests testing for HBV, HCV, and HIV as a component of the premarital health certificate [4]. In the current situation, when HBV infection still presents a serious health issue, this procedure creates an opportunity both to determine and further evaluate and monitor carrier cases, and to prevent the infection of their spouses and future children with the virus and the emergence of new carrier cases. The aim of this study was to test couples applying to a primary healthcare clinic for premarital testing for HBsAg and anti-HBs, and in case of HBsAg positivity, to introduce the prospective spouse with negative HBV markers into a rapid vaccination program on days 0, 7, and 21. We also aimed to compare the anti-HBs responses with the traditional vaccination scheme.

## 2. Materials and Methods

Blood samples were obtained for premarital testing for HBsAg and anti-HBs, and individuals sensitive to HBV whose future spouses were HBsAg positive were administered a three-dose rapid vaccination scheme on the 0th, 7th, and 21st days with the vaccine provided by the Ministry of Health (Euvax B). Blood samples were obtained at the 1st, 6th, and the 12th months after the last vaccination to check anti-HBs titers. All cases were also administered a booster dose at the 12th month. On the 13th and 14th months of vaccination, at least one month after the last dose of vaccine, one more blood sample was tested for anti-HBs titers. As the control group, 40 healthy volunteers, with similar age and gender who were negative for HBV markers, were administered three doses of the same vaccine at months 0, 1, and 6; control blood samples were obtained on the 45th day, and also 6 months and 5 years later. Statistical analysis was performed by Statcalc epi-info.

## 3. Results

Totally 1250 (625 couples) subjects were tested and 46 (3.6%) were found as HBsAg positive. 25 couples were already living together and had at least one child, in one of these households, both spouses were carriers. In terms of exposure to HBV of the spouses of other carrier cases, four had previously been exposed to HBV and immunized. Consequently, the remaining 40 subjects were admitted into the rapid vaccination program. The age range of the study group was 18 to 36, 22 were males and 18 females. The control group was consisted of 20 male and 20 female individuals whose ages ranged between 18 and 38.

One month after the completion of three vaccine doses, all cases in the study group had anti-HBs responses above the seroprotective level (10 mIU/mL). Of the control group of 20 individuals, only five cases showed protective anti-HBs response. Tests performed at the 6th month showed anti-HBs titers below the seroprotective level in one subject in the study group, while 17 persons in the control group had anti-HBs responses above the seroprotective level. 

The subject in the study group with an anti-HBs response below protective level was considered to be at high risk and administered a single booster dose vaccine, and blood tests performed one month after this vaccine showed a high anti-HBs titer (>100 mIU/mL). Blood tests at the 12th month demonstrated that 19 individuals in the study group and all subjects in the control group had anti-HBs responses above the protective level; blood samples tested one month after the last vaccine dose on month 12 in individuals in the study group showed high anti-HBs titers in all cases (Table 1). In the study group, anti-HBs responses above 10 IU/mL were statistically higher on the 45th day and in the 6th month when compared with the control group (*P* < 0.05). However this response was lower than the control group in the 12th month (*P* < 0.05). At the 5th-year control, both the study and the control group showed anti-HBs titers above 10 IU/mL.

## 4. Discussion

Hepatitis B vaccinations have been in use for approximately 20 years, produce high-titer antibody responses in adults and children, and are generally well tolerated [5–12]. Traditional vaccination scheme is three doses administered on the 0-1-6th months or four doses administered on the 0-1-2-12th months. Nevertheless, recent studies have investigated administration of three doses on days 0-7-21 or 0-10-21 with a booster dose in 12 months to subjects traveling to HBV endemic regions, those with an irregular former vaccine scheme, and subjects who need a rapid antibody response with positive results [13–15]. Bock et al. used three different vaccine schemes: 0-1-2nd day (group A), 0-14-28th day (group B), and 0-7-21st day (group C). Blood samples on day 28 showed that groups B and C had similar results, but significantly higher anti-HBs titers than group A; one month after the completion of three doses of vaccine, seroprotective anti-HBs titers in groups A, B, and C were 89%, 78.5%, and 76.4%, and on month 13, 95.8%, 98.9%, and 98.6%, respectively [16].

Marchou et al. vaccinated an adult group on days 0, 10, and 21 and found seroprotective anti-HBs responses of 40% on days 21, 91% on day 82, and 90% at the end of year 1 [17]. Another study by the same author investigated a scheme of vaccination on days 0-10-21 (group A) and months 0-1-2 (group B), with revaccination on month 12 in adults. In blood samples obtained one month after the completion of three doses, seroprotective anti-HBs responses were 70% in group A and 92% in group B; on month 12 prior to the administration of the booster dose, responses were 93% and 95%, respectively, and blood testing one month after the booster dose showed that seroprotection was provided in all cases [18].

Various studies concluded that a rapid vaccination scheme gave similar results with a traditional vaccination scheme, and anti-HBs response was maintained for at least 1 year [19, 20]. In our study, all cases administered a rapid vaccination scheme had developed a protective anti-HBs response at the end of month 1, while only five subjects in the group administered a traditional vaccination scheme had developed a protective anti-HBs response at the end of month 1. As subjects in the study group were couples applying for premarital testing and generally do not use condoms, an early anti-HBs response is important in this group. In the 6th month control, one subject in the study group had an anti-HBs response below the seroprotective level, while in the control group, 32 subjects had protective anti-HBs responses. On the 12th month, 19 subjects in the study group and all subjects in the control group had seroprotective anti-HBs responses, and one month after the 12th month vaccine administered to the study group, all cases had quite high anti-HBs titers. Although the number of cases included in the study group is not high, the rapid vaccination scheme can be favored in order to provide an early anti-HBs response in cases where premarital tests reveal HBsAg positivity in the prospective spouse. However, anti-HBs titers gradually diminish with this scheme of vaccination, and thus these subjects need to be closely monitored and revaccinated on month 12. The investigation of larger groups and the surveillance of subjects administered a rapid vaccination scheme in terms of anti-HBs titers in later years will provide further direction about the feasibility of this method.

## Figures and Tables

**Table 1:** Anti-HBs titers after the vaccination scheme in the study group and the control group.

	45th day		6th month		12th month		5th year	
	10 IU/mL	10 IU/mL	10 IU/mL	10 IU/mL	10 IU/mL	10 IU/mL	10 IU/mL	10 IU/mL
	under	over	under	over	under	over	under	over
Study group *n* = 40	—	40 (%100)	1 (%2.5)	39 (%97.5)	21 (%52.5)	19 (%47.5)	—	40 (%100)
Control group *n* = 40	35 (%87.5)	5 (%12.5)	8 (%20)	32 (%80)	—	40 (%40)	—	40 (%100)
Chi-square value	62.22				28.47		Na	
*P* value	0.000		0.028^#^		0.000^#^		Na	

^#^Fisher exact test result.

In the study group, anti-HBs responses above 10 IU/mL were statistically higher on the 45th day and in the 6th month when compared with the control group.

In the control group, anti-HBs responses above 10 IU/mL were statistically higher in the 12th month when compared with the study group.

Na: Not available.
